# Safety and Risk Assessment of No-Prescription Online Semaglutide Purchases

**DOI:** 10.1001/jamanetworkopen.2024.28280

**Published:** 2024-08-02

**Authors:** Amir Reza Ashraf, Tim K. Mackey, János Schmidt, Győző Kulcsár, Róbert György Vida, Jiawei Li, András Fittler

**Affiliations:** 1 Department of Pharmaceutics, Faculty of Pharmacy, University of Pécs, Pécs, Hungary; 2 Global Health Program, Department of Anthropology, University of California San Diego, La Jolla; 3 Global Health Policy and Data Institute, San Diego, California; 4 S-3 Research, San Diego, California; 5 Institute of Biochemistry and Medical Chemistry, Medical School, University of Pécs, Pécs, Hungary; 6 Department of Pharmaceutical Chemistry, Faculty of Pharmacy, University of Pécs, Pécs, Hungary

## Abstract

This qualitative study assesses the quality, amount of active ingredient, and characteristics associated with counterfeiting of semaglutide purchased from illegal online pharmacies without a prescription.

## Introduction

The popularity of branded semaglutide is surging, with widespread media coverage, viral social media exposure, and celebrity endorsements.^1^ Although Wegovy (Novo Nordisk) is approved for long-term weight management, Ozempic (Novo Nordisk) (only approved for type 2 diabetes) is often used off-label for this purpose. Global regulatory agencies, including the US Food and Drug Administration (FDA), European Medicines Agency, and World Health Organization (WHO), have warned about fake versions driven by patient demand, high cost, and shortages. Illegal online pharmacies, which operate without valid licenses and sell medicines like semaglutide without prescription, represent a consumer risk for ineffective and dangerous products.

## Methods

In this qualitative study, we conducted risk assessment of semaglutide online sourcing (Figure and eAppendix in Supplement 1). We followed the SRQR reporting guideline.

**Figure.**
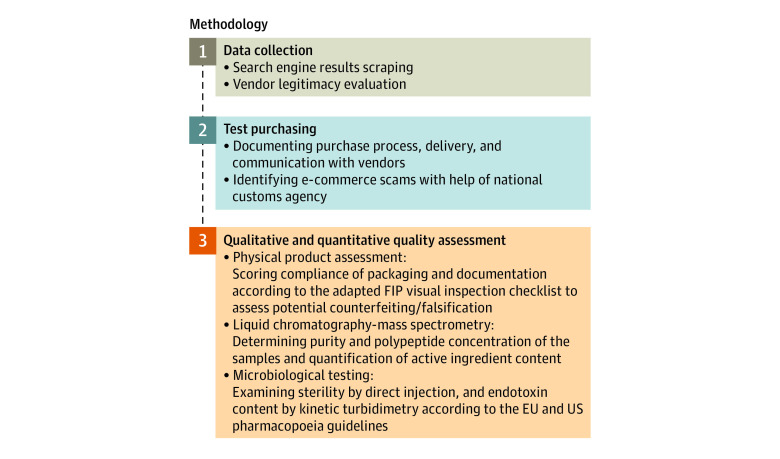
Summary of Study Methods Including Online Market Availability, Website Monitoring, Packaging Analysis, and Complex Product Quality Control Testing FIP indicates International Pharmaceutical Federation.

First, we conducted structured searches on Google and Bing to catalog websites advertising semaglutide without a prescription in July 2023. Websites meeting inclusion criteria were selected for a product test buy protocol.^2^ Two 0.25-mg per dose prefilled pens or equivalent semaglutide injection vials were ordered from each website. Upon product receipt, authors (A.R.A., and A.F.) used the International Pharmaceutical Federation’s (FIP) checklist for visual inspection to assess potential counterfeiting or falsification risks, compared with genuine Ozempic brand 1-mg semaglutide solution for injection in a prefilled pen.^3^ Products were then tested for quality, including sterility and microbiological contamination, according to European Pharmacopoeia and US Pharmacopeia guidelines. Quantification of active ingredients was performed using liquid chromatography–mass spectrometry (LC-MS). Test purchases and analytical testing were performed August from 2023 to March 2024.

## Results

Search engine monitoring generated 1080 hyperlinks, with 317 (29.35%) for online pharmacies. Nearly one-half (134 sites [42.27%]) belonged to illegal pharmacy operations; 763 links were websites not offering products for sale, including 615 news and informational websites and 148 telemedicine websites requiring consultation to obtain prescription before purchase.

Six online vendors classified as not recommended or rogue by LegitScript and/or National Association of Boards of Pharmacy and offering parenteral semaglutide products were included in test buys. Three websites offered prefilled 0.25-mg per dose semaglutide injection pens, and 3 sold vials of lyophilized semaglutide to be reconstituted to solution for injection (1-3 mg). All vendors referred to weight loss and obesity on their product page. Prices for the smallest dose and quantity ranged from US $113 to $360 (mean [SD], US $218.5 [$93.6]) (Table).

**Table.** Online Vendor Characteristics and Semaglutide Products Offered Online

Domain (location)^a^	Legit-Script verification	NABP category	Product form and dosage	Product price (shipping fee), $US^b^	Payment options	Prescription requirement	Assessment of patient health status before purchase	Communication of health-related benefits	Product packaging and labeling inspection^c^
semaspace.com (US)	NA	NR	Semaglutide vial (2 mg)	199/vial (30)	PayPal only	No	No health status required by seller	Yes, obesity	Substantial deficiencies (9/22 criteria met)
wieghtcrunchshop.com (Not listed)	Rogue	NR	Ozempic pen (0.25 mg) (not delivered)	190/1 pen (30)	Apple Pay, Google pay, Zelle, bank transfer, Osko, Bitcoin	No, “without prescription” highlighted	No health status required by seller	Yes, weight loss	Confirmed nondelivery scam operation
uschemlabs.com (Not listed)	Rogue	NA	Semaglutide vial (1 mg)	148.90/5 vials (25)	Credit card, CashApp, crypto-currencies	No	No health status or professional qualification requested	Yes, weight loss, blood glucose regulation	Substantial deficiencies (8/22 criteria met)
biotechpeptides.com (US)	Rogue	NR	Semaglutide vial (3 mg)	113/vial (0)	ACH, CashApp, Venmo, credit card	No	No health status or professional qualification requested	Yes, appetite, cardiovascular	Substantial deficiencies (8/22 criteria met)
puremedsonline.com (US)	Rogue	NR	Ozempic pen (0.25 mg) (not delivered)	300/2 pens (30)	PayPal, Zelle, Bitcoin	No, “without a doctors prescription now!!!” highlighted	No health status required by seller	Yes, diabetes, weight loss, cardiovascular	Confirmed nondelivery scam operation
genius-pharmacy.com (US)	Rogue	NR	Ozempic pen (0.25 mg) (not delivered)	360/2 pens (50)	Bitcoin, Zelle	No “No Rx required” highlighted	No health status required by seller	Yes, weight loss, diabetes	Confirmed nondelivery scam operation

Abbreviations: ACH, Automated Clearing House; NA, not applicable; NABP, National Association of Boards of Pharmacy; NR, not recommended.

^a^ Purported location listed on website.

^b^ Prices represent the smallest quantity offered for sale.

^c^ Each International Pharmaceutical Federation criterion was scored as present (1) or absent (0) for a total score range of 0 to 22, with lower scores indicating higher risk.

Test purchases were confirmed via email and WhatsApp. Of 6 products purchased, only 3 were received. Three vendors selling Ozempic injections engaged in nondelivery scams requesting extra payments (range, US $650-$1200) to purportedly clear customs, confirmed as fraudulent by customs agencies. Although genuine Ozempic scored the full 22 points on the FIP checklist, test purchased products scored 8 or 9 with clear discrepancies in regulatory registration information, accurate labeling, and evidence products were likely unregistered or unlicensed.

Upon quality testing, one sample had elevated presence of endotoxin (8.95 EU/mg) indicating possible contamination, although no viable microorganisms were detected. LC-MS revealed the presence of semaglutide in all samples, but with considerably lower purity levels (7%-14% vs advertised 99%). The measured semaglutide content substantially exceeded the labeled amount in each sample by 29% to 39%, meaning that users could receive up to 39% more semaglutide per injection. These risk factors indicate likely falsification that does not meet legitimate product quality standards.

## Discussion

This qualitative study found that semaglutide products are actively being sold without prescription by illegal online pharmacies, with vendors shipping unregistered and falsified products. Two websites evaluated were sent FDA warning letters for unlawful sale of unapproved and misbranded semaglutide.^4,5^ US poison centers have reported a 1500% increase in calls related to semaglutide, highlighting the need for enhanced pharmacovigilance including for online sourcing harms.^6^ Study limitations include limited sample of products tested due to nondelivery scams. Furthermore, although tested products represent some accessible semaglutide products sold online, higher priced offerings were excluded, limiting generalizability of the findings.

## Notes

Supplement 1. **eAppendix.** Description of Methods

Supplement 2.
Data Sharing Statement
