# Supplementary material for: Safety and Risk Assessment of No-Prescription Online Semaglutide Purchases
Source: JAMA Netw Open. 2024 Aug 2;7(8):e2428280. doi: 10.1001/jamanetworkopen.2024.28280 (PMC11297364; doi:10.1001/jamanetworkopen.2024.28280)
Supplement: Supplement 1. — eAppendix. Description of Methods [file jamanetwopen-e2428280-s001.pdf]

Supplemental Online Content

Ashraf AR, Mackey TK, Schmidt J, et al. Safety and risk assessment of no-prescription online semaglutide purchases. *JAMA Netw Open*. 2024;7(8):e2428280.
doi:10.1001/jamanetworkopen.2024.28280

eAppendix. Description of Methods

This supplemental material has been provided by the authors to give readers additional information about their work.

© 2024 Ashraf AR et al. *JAMA Network Open.*

eAppendix. Description of Methods

1.1. Structured searches on Google and Bing using automated web crawling

Google and Bing search engines were queried for "buy [Proprietary Name/API]" and "buy [Proprietary Name/API] without prescription" keyword combinations to record links generated from search engine result pages for keywords Ozempic, Wegovy and semaglutide. These keywords were chosen based on relevance to consumer awareness about semaglutide-related search terms that were also identified through Google Trends analysis at the time of study preparation. Data collection was performed in July 2023 using an automated web crawling program developed in the Python programming language using the package Selenium, scraping search engine results with all web browser cookies data, cached data, and search history deleted prior to each search. Results of returned hyperlinks were then generated in JSON format, which was then exported into .xlsx format for manual review and annotation.

1.2. Website selection and assessment criteria

Previously published research has indicated that individuals tend to click on links suggested on the first results page during their search, with the first 10 links receiving the majority of the organic user click rate, and the calculated cumulative click through rates after the first 30 results are negligible. Therefore, the first 30 results for each search query performed using Google and Bing search engines were recorded and examined (1080 websites in total), providing a good representation of website links that a consumer would be exposed to through the two most popular search engines used globally. Websites were manually assessed by two authors (ARA and AF) based on the study inclusion criteria.

1.3. Online seller legitimacy classification

Websites were reviewed manually by two authors (ARA and AF) to confirm selling of injectable semaglutide containing products without a prescription and assessed for legitimacy using LegitScript and the National Association of Boards of Pharmacy (NABP) databases, discrepancies were resolved through discussion to reach consensus. All domains were categorized as illegal pharmacies if classified as "rogue" by LegitScript and/or categorized as "not recommended" according to the NABP Safe Pharmacy verification database. LegitScript is a private company that monitors and classifies online pharmacies based on their compliance with applicable laws and regulations. Pharmacies are classified as "rogue" if they are operating out of compliance, illegally or fraudulently. NABP is a nonprofit organization that supports and works with the U.S. state boards of pharmacy and maintains a list of websites that are reviewed for possible fraudulent and unsafe prescription medication sales online. Unverified pharmacy websites, whose domains were not listed in these databases, underwent a thorough manual evaluation to determine legitimacy by two authors independently.

2. Website content analysis and inclusion criteria for test purchases

Websites inaccessible at time of evaluation, referral sites not offering products directly to customers, duplicate content websites (different domains with identical content), ones requiring valid medical prescription before purchase were excluded, vendors delivering products to Hungary or US, and sites selling products for up to 200 USD per item were included in detailed content analysis. Six were selected[1] for test purchases, two 0.25 mg ampules/doses

---

[1] Links to digital archives of the selected illicit online pharmacies analyzed in this study, hosted on the Internet Archive Wayback Machine. Links are listed in the same order as they appear in the manuscript Table 1.
https://web.archive.org/web/20230529141701/https://semaspace.com/

© 2024 Ashraf AR et al. *JAMA Network Open.*

or an equivalent product was ordered from each vendor to simulate the initial 2-week therapy regimen of a patient. Credit cards, PayPal, or bank transfers were the preferred payment options. The chemical and microbiological analyses were to be conducted in Hungary. Therefore, it was deemed preferable for shipments to be directly dispatched to Hungary. In instances where direct delivery to Hungary was not offered by the vendor, the parcels were initially sent to California, USA, and subsequently forwarded to Hungary.

3. Packaging risk assessment

International Pharmaceutical Federation's (FIP) checklist for visual inspection of medicines, which was initially designed to aid healthcare workers in identification of substandard and falsified products, was adapted for the evaluation of packaging and labelling of delivered products. The checklist evaluates 22 criteria related to packaging integrity, labeling, and regulatory compliance, with each criterion scored as present (1) or absent (0) for a total score range of 0-22. Lower scores indicate higher risk of substandard and falsified medicines. The visual inspection involved observation and photo documentation of the primary and secondary packaging, leaflets and labelling. Scoring discrepancies were resolved through discussion to reach consensus.

4. Sterility and microbiological contamination

Sterility and microbiological contamination assessment tests were performed on lyophilized peptide samples obtained from Semaspace, Biotech Peptides, and US Chem Labs to assess the product quality. Testing was performed at the ISO 14644-1 certified microbiology laboratory of PharmaValid Ltd. in Budapest, Hungary. Sterility testing was done using direct injection technique according to European Pharmacopoeia (Ph. Eur. 11.0 2023 2.6.1) and United States Pharmacopeia (USP-NF2023 ISSUE 2 <71>) guidelines, and bacterial endotoxin content measurement was performed via kinetic turbidimetry technique according to the European Pharmacopoeia (Ph. Eur. 11.0 2023 2.6.14) and United States Pharmacopeia (USP-NF 2023 ISSUE 2 <85>) guidelines.

5. Quantification of active ingredient using liquid chromatography/mass spectrometry

Stock solutions of the standard and the polypeptide samples were prepared in methanol. The working solutions were diluted using water/acetonitrile/formic acid (49/49/2, v/v/v). Chromatographic separation was performed using a Thermo Ultimate 3000 UHPLC™ system (Thermo Fisher Scientific, Waltham, MA, USA) with a Luna Omega PS-C18 reversed-phase column (1.6 µm, 2.1 mm × 150 mm i.d.) from Phenomenex (Torrance, CA, USA). Data-dependent mass spectrometric acquisition was performed with a Bruker Maxis 4G UHR-QTOF instrument (Bruker Daltonics, Bremen, Germany). The mass spectrometer was operated in positive ion mode, and the scanning range was set to 300–2200 m/z.

6. Ethical approval

The institutional review board at the University of Pécs reviewed the study protocol and granted a waiver of ethical approval as the study did not involve human participants and utilized only

---

https://web.archive.org/web/20230724173122/https://www.wieghtcrunchshop.com/
https://web.archive.org/web/20230623211355/https://www.uschemlabs.com/
https://web.archive.org/web/20230620220903/https://biotechpeptides.com/
https://web.archive.org/web/20230508160023/https://www.puremedsonline.com/
https://web.archive.org/web/20230117213347/https://genius-pharmacy.com/

© 2024 Ashraf AR et al. *JAMA Network Open.*

publicly available online information. We aimed to provide a transparent and comprehensive account of our research process and findings. Descriptive statistics were performed using Excel (Microsoft 365 MSO) to summarize website characteristics, visual inspection scores, and chemical analysis results.

© 2024 Ashraf AR et al. *JAMA Network Open.*
