# Supplementary material for: Safety and Risk Assessment of No-Prescription Online Semaglutide Purchases
Source: JAMA Netw Open. 2024 Aug 2;7(8):e2428280. doi: 10.1001/jamanetworkopen.2024.28280 (PMC11297364; doi:10.1001/jamanetworkopen.2024.28280)
Supplement: Supplement 2. — Data Sharing Statement [file jamanetwopen-e2428280-s002.pdf]

Data Sharing Statement

Ashraf. Safety and Risk Assessment of No-Prescription Online Semaglutide Purchases. *JAMA Netw Open*. Published August 02, 2024. doi:10.1001/jamanetworkopen.2024.28280

Data
Data available: Yes
Data types: Data (not involving human participants)
How to access data: The data available from this study includes detailed methodologies of search engine result scraping techniques used to extract organic and paid results from Google and Bing, a dataset with links to the evaluated online pharmacies, website legitimacy evaluation methods, screenshots of the websites involved in the study and the ordering processes, records of communications with sellers, product evaluation checklist and the methods and results of microbiological and LC/MS testing of the products. Data will be made available upon request to the corresponding author via email. Interested parties are encouraged to specify the type of data they are requesting and the intended use in their inquiry. The corresponding author will provide the requested data along with any conditions for their use. To ensure the protection of privacy and confidentiality, some data including communication with the sellers and payment information may be redacted or anonymized as necessary. Requests for data should be sent to Tim K Mackey, MAS, PhD to the following email address: tkmackey@ucsd.edu
When available: With publication

Supporting Documents
Document types: None

Additional Information
Who can access the data: Anyone requesting the data
Types of analyses: For any research purpose or a specified research purpose
Mechanisms of data availability: Without investigator support
